# Miracles of war

**DOI:** 10.3325/cmj.2011.52.436

**Published:** 2011-06

**Authors:** Zoran Aleksijević, Agneza Aleksijević

**Affiliations:** Surgery Ward, Vukovar Medical Center, Vukovar, Croatia

Zoran Aleksijević was born in 1957 in Montenegro. He completed medical studies and residency in Novi Sad and worked as a physician in the Vukovar Medical Center since 1982, and as a specialist in orthopedics and trauma surgery since November 1990.

Agneza Aleksijević was born in 1962. She worked in the Vukovar Medical Center as a theater nurse.

## Accommodation and facilities

The Surgery Ward was located on the third floor, under the roof. After the first attacks by the Yugoslav Federal Army (YFA) we moved to the basement, where outpatient offices were located. We set up operating theaters in available rooms. The table for plaster application and examination table in the x-ray room served for surgery. The table moved and shifted during surgeries, and we had to hold it with the help of nurses. We considered outer walls dangerous and lowered heads when the shells fell. Later, we got used to it. The patients were accommodated in the safest place – basement corridors, two walls away from the outside. One or two patients would share a single bed, and another one would sleep on a mattress below the bed. We slept where and when we could, on a chair, in a corner. I slept with my wife in a tiny room (2 m^2^), previously used a change room for patients to prepare for examination. It was impossible to stretch and rats ran over us – it was so crowded that there was no place even for them. We tried to get rid of them with baits made from a mixture of flour and plaster of Paris.

Soon, the glass windows broke and we closed them with planks and plastic sheets. It was dark in the basement and we lost orientation in time.

Once, YFA planes dropped a heavy bomb (we called it a “sow” because it could weigh 250-500 kg) without a detonator, in order to damage the foundations of the hospital. They did not succeed.

Heavy artillery fire never ceased; we had an impression the floor waved like a ship. We learned to distinguish various heavy artillery shells by sound.

After an attack, the heating system collapsed. The stoker was killed by a cannon shell in his boiler room. It grew very cold.

Electricity went out too, but we used three electric generators that worked alternately until only one was left. Surgeries were performed under the light of battery lamps, candles and improvised oil lamps that were held by Agneza and other nurses.

Families of the employees were also in the hospital shelter.

## Food and nutrition

We soon felt a shortage of food. In the beginning, we would get a rusk and a half cup of tea for breakfast. Hard rusks made wounds on our mouths and gums. Later, buns the size of a small saucer, hard and underdone, were baked without yeast. Lunch consisted of a soup (made of browning flour on oil and water) without salt. We shared all food with patients. Croatian National guardsmen would occasionally catch a pig, which was shared by more than 600 people. We shared an occasional pack of biscuits or bottle of beer with patients. Guardsmen would bring all the food they found first to the hospital for the wounded and medical staff. We all lost up to 10 kg in weight.

Nobody in Vukovar had enough food or water. People were dying in their cellars from hunger and thirst. As a local department store still worked from 8-10 am, people went out to buy food risking sniping rifle death.

## Surgeons and their patients

After the massacre in Borovo Selo on May 2, 1991, it was difficult to decide whether to spend the night in the hospital or return to our homes. Official working hours were 7 am to 10 pm After the first heavy attacks, we stayed in the hospital for 88 days.

Dr Vesna Bosanac took care of all of us. She was tireless. She made telephone calls throughout the day and tried to contact everybody who could help us. She was a good organizer. She tried to save the wounded and the hospital staff from the army until the last moment. We cannot understand how she could be accused of any misconduct.

Five surgeons worked in the Surgery Ward. Three general surgeons, few anesthesiologists and nurses did not take any active part in our work, but nursed five reservists and several civilians. They would go to the anti-atom bomb shelter during attacks. Thoracic and abdominal surgeries were performed by dr. Njavro, and the rest by me. Agneza and one other nurse assisted during the surgeries. A specialist for child surgery from Zagreb was also with us.

Anesthesiologists were very skilful and used anesthetics economically and carefully. Minor surgeries were performed in local anesthesia with 1% xylocaine. Most of the time, the surgeries were performed under spinal or block anesthesia. Patients did not feel pain, but they were conscious. They received a sedative (equivalent of valium) after the surgery and, exhausted, fell asleep right away. General anesthesia was only used in cases of thoracic and abdominal surgery.

All wounded patients were very exhausted, starved, wet and anemic (1.7-1.8 × 10^12^ erythrocytes per liter). This was the only test we could do until the very end, but we managed even without laboratory results. Hospital staff donated blood for the wounded. When wounded guardsmen arrived in the hospital, they would sleep for 3-4 days. We would admit 25-90 casualties daily. Soldiers drove their wounded comrades in the dark and without lights through the wrecked streets of Vukovar. Vans broke when they drove over holes made by ‘sow’ bombs. The drivers were very brave. They would sound a horn once shortly upon their arrival in front of the hospital. We would run to the Emergency entrance and examined patients in the van. Anesthesiologist would insert endotracheal tubes if necessary. Agneza and other nurses helped to carry out the wounded. It was dangerous because this entrance was a constant target of sniping-rifle fire. Agneza helped despite her great fear. Once a detonation threw her backward a few meters, under the table of Dr Bosanac, whose room was located opposite of this entrance.

We helped all the wounded and treated all those who needed medical care. Usually we did not see what uniforms they wore, as the patients were already prepared for a surgery.

We kept records on the wounded admitted to the hospital, performed surgeries and the deceased. Some cases were recorded on photographs. YFA soldiers took away all the records on Tuesday, November 19, 1991. We were forbidden to keep anything. Agneza and I secretly took my camera with a few pictures taken and her hair dryer.

In total, we treated 2500 wounded and I performed 1200 surgeries. I placed more than 100 external fixators. When we ran out of them, Agneza suggested that we find patients with healed fractures, treat them further with plaster and use their fixators for the newly wounded. We applied our modification of a splint for shotgun wounds of the limbs, which consisted of a circular plaster dressing with openings for the wounds to make care possible. In the beginning, we diagnosed fractures and controlled the position of fragments with the help of x-ray machines. However, power supplies could not provide enough electricity, so we diagnosed fractures by inspection or during the surgery. We tried to assure correct position of fragments. Electrocauteries could not be used and we had problems with hemostasis. We successfully saved injured limbs, even when an amputation was indicated according to the surgical standards. Because we were with our patients all the time, we closely monitored their condition. Despite everything, amputations were sometimes unavoidable. Our patients absolutely trusted us, they believed us when we told them that an amputation was necessary. I performed several amputations at the hip level. In one case of gaseous gangrene, I had to remove the arm, the shoulder bone and the pectoral muscle. On another occasion, we were not aware of the extent of injuries during the surgery. After the removal of sheets, there was very little left of a man who underwent several amputations and tissue excision. Agneza is even now amazed how we managed to have our meal after performing an amputation. We watched over patients carefully, often held their hand or laid our hands on their shoulders. It meant a lot to them. Agneza would come and warn me if a patient was feeling worse. We knew their names or nicknames. We successfully sutured blood vessels. Injured nerves were left for later surgeries. Wounds were extensive, with vast soft tissue and bone defects. Entrance wounds were small and the exit wounds large. Tissue was often turned to pulp as a consequence of high speed projectiles. Wounds of the genitalia were also frequently encountered. A man suffered extensive napalm bomb burns on his head, neck, chest and hands. Terrible smell spread through the hospital. He developed edemas after a short time and died. The wounds inflicted by tread-mines (so-called tin-cans) were particularly ugly. Soldiers told us they found human parts glued to the walls in a geriatric home destroyed by rockets. An old woman they brought was literally cut in two pieces.

Our soldiers were incredibly enduring. One of them was brought in after surviving for five days in a cornfield, heavily wounded, one arm almost torn off. He lost consciousness, crawled, fell in ditches, tried to kill himself because of great pain and was finally brought in the hospital. Debridement of the wounds was performed but the sutures were not laid. Antitetanus prophylaxis was obligatory. Compresses soaked in 3% saline were put on soiled wounds, and those soaked in 10% saline on clean ones. Necrectomy was performed regularly. The wounds mostly healed well, with good and healthy granulations.

Antibiotics were not abundant. Physicians without Borders could not bring us anything. We got two packs of antibiotics from Germany, a few packs of each antibiotic group, for both oral and parenteral application. We prescribed them on the basis of clinical assessment. The laboratory was destroyed very early and microbiological analyses were not available. Infusion solutions were scarce, plasma not available, and the pharmacy personnel distilled and re-boiled rain or well water. Four out of nine patients with gaseous gangrene died. Infections with pyocyaneus were common. Antiseptics and solutions for disinfection, mostly based on iodine (Povidone), were used sparingly. Every drop was precious. Nurses wiped the instruments, washed them with some water and disinfection solution and sterilized them in hot air. Instruments used for attending and re-dressings in patients with gaseous gangrene were kept and disinfected separately. Despite extremely difficult conditions, we did not have any infections after chest or abdomen surgery. A case of primary pyothorax was observed. Good results of the operations despite the lack of water and the proper sanitation inspired Dr Njavro to swear he would never do the twenty-minute hand wash once the war ended.

The clothes we wore were the only we had. After a warehouse was damaged, our soldiers brought us sweat suits. Patients and hospital staff had the same. Nurses wore several layers of clothing for surgeries. Coats were changed twice a month. I even operated in my sweat suit. We ran out of masks and tied elastic bandages over mouth and nose. We kept our surgical caps in a pocket. Toward the end, our clothes got very dirty and torn. Blankets and sheets could not be changed. Patients could not be washed. Hands could not be washed. We wore double gloves, rubber and plastic underneath. I sometimes operated without gloves.

In the beginning, the dead were buried in the local cemetery, then in common graves and at the stadium. The graves were covered with some earth which was soon washed away by rain, and the corpses taken apart by pigs and dogs. We kept records of all the deceased. When we ran out of coffins, the corpses were put in black plastic bags and finally in very dirty blankets. Corpses with tied hands and bandage around jaw and head, with numbers on their chest appeared on television. These were not people slaughtered by Croatians, but those who died in the hospital or were brought in dead.

In rare moments of the free time Dr Njavro and I made plans of hospital renovation after the war. We arranged everything. We even named it after one of our heroes. We listened to the news on Radio Osijek broadcasting the Croatian radio program. National guards would also inform us. It turned out they kept some information from us, having no courage to reveal the truth. Until the end, we were sure that Vukovar would not fall. One day, during a surgery, Agneza whispered in my ear that Vukovar had fallen. She could not bear it. At that moment, I did not know what to do next. On Monday, November 18, 1991, the YFA and the Serbian paramilitary forces entered the hospital. They forbade us to work and take care of the patients. We had to stay in our rooms; it was dangerous even to go to the washroom. All medical documentation and records were taken away and the YFA demanded new lists of patients from our clerk over and over again. Next day, one of our colleagues was heavily beaten and could not even stand. A wounded patient was killed immediately in front of the hospital. Our hospital employed 1100 personnel before the war. At the end, only 300 of us were left. When the YFA occupied our hospital, approximately 45 persons, family members of the employees, were in the anti-atom bomb shelter, with 700 wounded, among whom 213 or 236 (I do not recall which number is correct) were severely injured. Recovering patients were located in 3-4 other shelters in the town. When we left the hospital on Tuesday, we were sure we were going to be killed. Through a small and dirty window of the transport vehicle I saw, for the first time, the extent of the destruction in Vukovar. A huge hole was in the place of four apartments in the building where we lived, including ours. We were accepted quite well in Sremska Mitrovica, especially when they heard I was from Montenegro. We were allowed to drink water, and were offered warmed cans of beans and beer. The next day, another group of the YFA soldiers came, and they threatened to cut our throats. When we finally reached Đakovo in Croatia, we bought new clothes, changed in the store and threw the torn and soiled clothes away.

